# A novel FOXM1–BCL2A1 axis determines unfavorable response to venetoclax in AML

**DOI:** 10.1016/j.jbc.2025.108240

**Published:** 2025-01-27

**Authors:** Sanjeev Raghuwanshi, Ahmed Magdy, Nissim Hay, Andrei Gartel

**Affiliations:** 1 University of Illinois at Chicago, Department of Medicine, Chicago, Illinois, USA; 2 University of Illinois at Chicago, Department of Biochemistry and Molecular Genetics, Chicago, Illinois, USA

**Keywords:** AML, apoptosis, BCL2A1, FOXM1, venetoclax

## Abstract

Forkhead box M1 (FOXM1), a Forkhead family transcription factor, is often overexpressed in a variety of human cancers, including acute myeloid leukemia (AML), and is strongly associated with therapy resistance and unfavorable outcomes. In AML with *NPM1* mutations, NPM1–FOXM1 complex sequesters FOXM1 in the cytoplasm and confers favorable treatment outcomes for AML patients because of FOXM1 inactivation. Inhibition of FOXM1 in AML cell lines and animal models of AML sensitizes AML cells to the BCL2 inhibitor, venetoclax. In a recent study, the upregulation of the BCL2-family protein, BCL2A1, conferred resistance to venetoclax and multiple venetoclax combinations. In this study, we investigated the FOXM1–BCL2A1 axis and determined that FOXM1 specifically inhibits venetoclax-induced apoptosis in AML *via* upregulation of BCL2A1. The knockdown of BCL2A1 in AML in the presence of high levels of FOXM1 led to sensitization of AML cells to venetoclax, suggesting that BCL2A1 is a major target of FOXM1 responsible for resistance to venetoclax. Venetoclax in combination with FOXM1 inhibitor STL001 inhibited BCL2A1 and circumvented venetoclax resistance. Pharmacological inhibition of the FOXM1–BCL2A1 axis represents a therapeutic strategy to sensitize AML cells to venetoclax-induced apoptosis.

Acute myeloid leukemia (AML) is an extremely aggressive and heterogeneous disease driven by recurrent cytogenetic abnormalities and hotspot mutations (1, 2). Notably, AML has a poor prognosis; 10 to 40% of younger and 40 to 60% of above 60 years AML patients are primarily refractory to AML induction therapy and failed to achieve complete remission (1, 2). Augmentation of antiapoptotic signaling, primarily driven by the upregulation of prosurvival BCL2 family proteins, is a key characteristic of cancer cells and has an essential role in mediating AML survival and chemoresistance (3).

Apoptosis can be triggered by a variety of extracellular (extrinsic) or intracellular (intrinsic) stimuli; in recent years, the molecular machinery driving the intrinsic and extrinsic apoptotic pathways has been elucidated (4). Intrinsic apoptosis relies on the balance between proapoptotic and antiapoptotic proteins inducing mitochondrial outer membrane permeabilization (MOMP), ultimately leading to caspase-dependent cell death. MOMP is mainly regulated and balanced by protein-protein interactions among members of the *BCL2* gene family (3, 4). Different members of the BCL2 family contain at least one of four relatively conserved BCL2 homology motifs (BH1–BH4) (3, 5). Antiapoptotic proteins, including BCL2 and four relatives (BCL2L1 [Bcl-xL], BCL2L2 [BCL-w], BCL2A1, and MCL1) sequester proapoptotic proteins by binding to its BH3 motifs and promote cell survival (3, 5). Proapoptotic members instead elicit cell death and consist of BH3-only proteins and effector proteins, BAK and BAX, which have BH1-4 motifs. BH3-only proteins, containing a single BH3 domain, act as sensitizers (BAD, BIK, HRK, and NOXA) or activators (BIM, BID, and PUMA) of apoptosis (4, 5). BH3-only sensitizer proteins do not directly activate effectors (BAK and BAX); instead, they function by binding to and neutralizing BCL-2 antiapoptotic protein, thereby releasing bound BAK and BAX effectors or BH3-only activator protein (6). Upon activation by bound BH3–only activator proteins, BAK/BAX protein oligomerizes, leading to MOMP and initiation of cytochrome *c*-mediated intrinsic apoptosis (5).

The prosurvival BCL2 proteins are often upregulated in AML cells to avoid apoptosis (7, 8, 9). Therefore, targeting antiapoptotic BCL2 proteins has long been an attractive therapeutic strategy to treat AML and other hematological malignancies. Since 2017, the Food and Drug Administration approved several targeted drugs for AML, including BCL2 inhibitor venetoclax (10, 11). The combination of venetoclax with low-dose hypomethylation agents exerted a favorable overall response in about 70% of older patients with *de novo* AML (11, 12). However, it was only modestly effective in a cohort of relapse/refractory AML patients as monotherapy (CR/CRi rates 19%) or in combination with hypomethylation agents (CR/CRi rates 54%) (13, 14). In most patients, acquired resistance following venetoclax treatment results in treatment failure. The mechanism of resistance is not immediately clear, but recent reports have highlighted that the AML cells may not depend on BCL2 for their survival or dependency on other prosurvival BCL2 family proteins may evolve during tumor progression and after therapy (5). The upregulation of other prosurvival BCL2 family proteins, including BCL2A1, MCL1, BCL-xL, and BCL-w, is one of the main determinants of resistance to venetoclax-induced apoptosis (9, 15, 16, 17, 18, 19).

Cytoplasmic NPM1 carried mutations (*NPM*^*mut*^) identified in a fourth of AML cases and are highly deterministic of treatment response (20). The favorable responses of *NPM*^*mut*^ to induction chemotherapy (20) or BCL2-targeted therapy (21) have been confirmed by multiple studies. We have identified NPM1 as a binding partner of FOXM1 and a critical determinant of FOXM1 cellular localization (22, 23). FOXM1 was identified as a possible candidate protein that can explain the vulnerability of *NPM*^*mut*^ AML cells to cytotoxic or BCL2-targeted therapy.

FOXM1 is a Forkhead family transcription factor that is well known to promote all the hallmarks of cancer. We have previously demonstrated that FOXM1 knockdown (KD) induces sensitivity to cytotoxic or BCL2-targeted therapy in several human cancers, including AML (24, 25, 26). A cancer-wide meta-analysis identified the FOXM1-regulatory network as a major predictor of adverse outcomes (27). Recently, we have performed a correlation study between a FOXM1 transcriptional signature and *NPM1* mutational status and its independent prognostic significance using the OHSU Beat AML database (25). Interestingly, for the first time, we showed low FOXM1 activity in AML patients with *NPM*^*mut*^ and that low FOXM1 activity is an independent predictor of chemotherapy response and disease-free survival for AML patients (25).

In the current study, we first sought to assess the expression of apoptosis regulatory genes modulating venetoclax efficacy using RNA-Seq data derived from KG-1 cells with stable shRNA–mediated FOXM1-KD. We found a positive correlation between *FOXM1* and *BCL2A1* expression, and their suppression synergized with venetoclax to induce apoptosis in AML cells. FOXM1 overexpression reduced venetoclax sensitivity of AML by stabilizing BCL2A1; this effect was recapitulated by BCL2A1 overexpression with no significant change in FOXM1 expression levels. Further, pharmacological inhibition of FOXM1 using STL001 confirmed that FOXM1 determines resistance to venetoclax-induced apoptosis through the regulation of BCL2A1.

## Results and discussion

**Figure 1:**
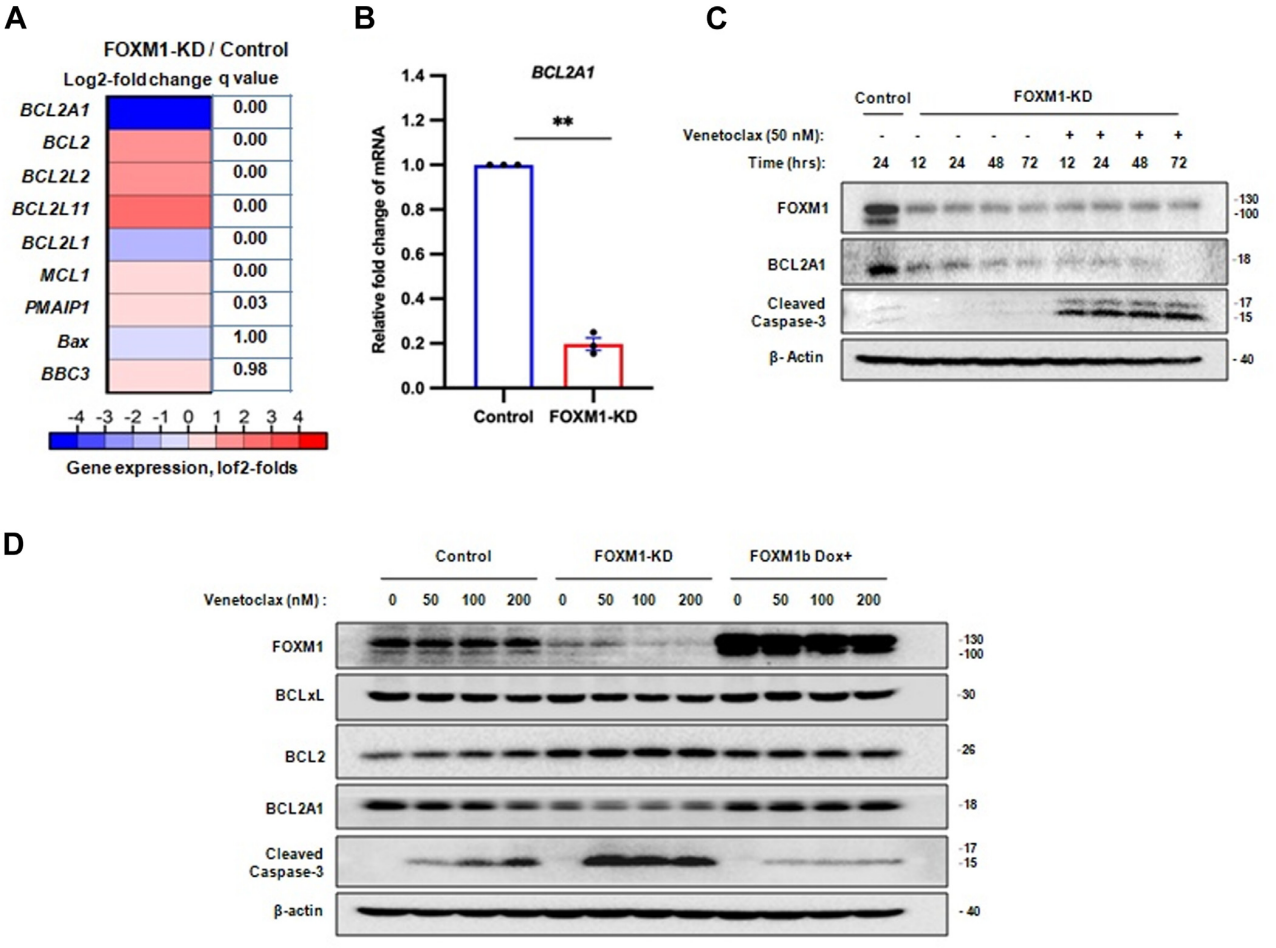
**FOXM1 confers venetoclax resistance by stabilizing BCL2A1 expression levels.***A*, expression changes in genes contributing to venetoclax resistance in AML. The heatmap displays log2-transformed differences in gene expression levels between FOXM1-KD and untreated control KG-1 cells. Data were exported from our previously published RNA-Seq analysis in AML cell line KG-1 (https://doi.org/10.3389/fonc.2021.696532). *B*, relative fold change of *BCL2A1* mRNA in KG-1-FOXM1-KD compared with control scramble vector–transduced KG-1 cells. *GAPDH* was used as an internal loading control. Data presented as mean ± SEM (n = 3 biologically independent replicates; ∗∗*p* < 0.002), for comparison two-tailed Student’s *t-*test was used. *C*, KG-1 cells with stable shRNA–mediated FOXM1-KD grown in the absence or the presence of venetoclax (50 nM) at indicated time points and compared with control KG-1 cells. In all cases, total protein samples were obtained from cells immediately after treatment and analyzed for FOXM1, BCL2A1, and cleaved caspase-3 levels *via* immunoblotting, and β-actin was used as an internal loading control. The blot shown is representative of two independent experiments with consistent results. *D*, KG-1 cells with stable shRNA–mediated FOXM1-KD, doxycycline (Dox)-inducible FOXM1b overexpression, or control vector were treated with indicated concentrations of venetoclax for 24 h. In all cases, total protein samples were obtained from cells immediately after treatment and analyzed for FOXM1, Bcl-XL, BCL2, BCL2A1, and cleaved caspase-3 levels *via* immunoblotting, and β-actin was used as internal loading control. The blot shown is representative of two independent experiments with consistent results. AML, acute myeloid leukemia; FOXM1, Forkhead box M1; KD, knockdown.

Given that FOXM1 is a master transcriptional regulator that is strongly associated with a reduced response to therapy in several cancers including AML, we aimed to understand how FOXM1 reduces the sensitivity of AML cells to venetoclax. Our laboratory previously performed RNA-Seq analysis in KG-1 cells with stable shRNA–mediated FOXM1-KD (28). We evaluated the expression changes of *BCL2* family genes modulating venetoclax efficacy (9, 15) and identified *BCL2A1* as a gene with the most intriguing pattern of expression changes associated with FOXM1 levels (Fig. 1*A*). Indeed, a similar expression pattern of *BCL2A1* was recorded in quantitative RT–PCR (qRT–PCR) analysis, *BCL2A1* mRNA levels were ∼7-fold lower in FOXM1-KD compared with scramble control KG-1 cells (Fig. 1*B*). We further conducted Western blot analyses of BCL2A1 protein from KG-1-FOXM1-KD cells grown in the absence or the presence of venetoclax (50 nM) for various time points. We observed that FOXM1-KD cells express very low levels of BCL2A1 protein at all indicated time points when compared with nontargeting control cells (Fig. 1*C*). We also found that FOXM1-KD cells treated with venetoclax as low as 50 nM led to potent induction of apoptosis, indicated by caspase-3 cleavage initiated within 12 h of treatment (Fig. 1*C*). This finding is consistent with earlier reports of BCL2A1 being a crucial regulator of venetoclax sensitivity in AML (9), highlights a positive association between FOXM1 and BCL2A1 expression, and shows that FOXM1 and BCL2A1 suppression synergized with venetoclax to induce apoptosis in AML cells.

To confirm this correlation and to test how the expression of these genes is linked to venetoclax response, we performed immunoblot analysis of KG-1 cells with stable shRNA–mediated FOXM1-KD, doxycycline (Dox)-inducible FOXM1 overexpression, or control vector after treatment with different concentrations of venetoclax. We observed that KG-1 cells with FOXM1b overexpression (Dox+) demonstrated higher FOXM1 and similar levels of BCL2A1 expression as compared with control vector cells; notably, the expression levels of BCL2A1 were stable across different concentrations of venetoclax as compared with control and were higher than in KG-1-FOXM1-KD cells (Fig. 1*D*). This prompted us to further investigate the correlation between FOXM1-mediated BCL2A1 expression and venetoclax sensitivity. While venetoclax concentrations as low as 50 nM readily induced apoptosis in FOXM1-KD cells, less apoptosis occurred in control vector cells at very high concentrations (200 nM), as indicated by caspase-3 cleavage (Fig. 1*D*). Interestingly, KG-1 cells with FOXM1-Dox+ demonstrated a stable BCL2A1 level and no apoptosis across different concentrations of venetoclax, even at a high dose (200 nM; Fig. 1*D*). These results show a positive correlation between FOXM1-mediated BCL2A1 expression and venetoclax resistance, confirming that BCL2A1 is regulated by FOXM1 (Fig. 1*D*).

**Figure 2:**
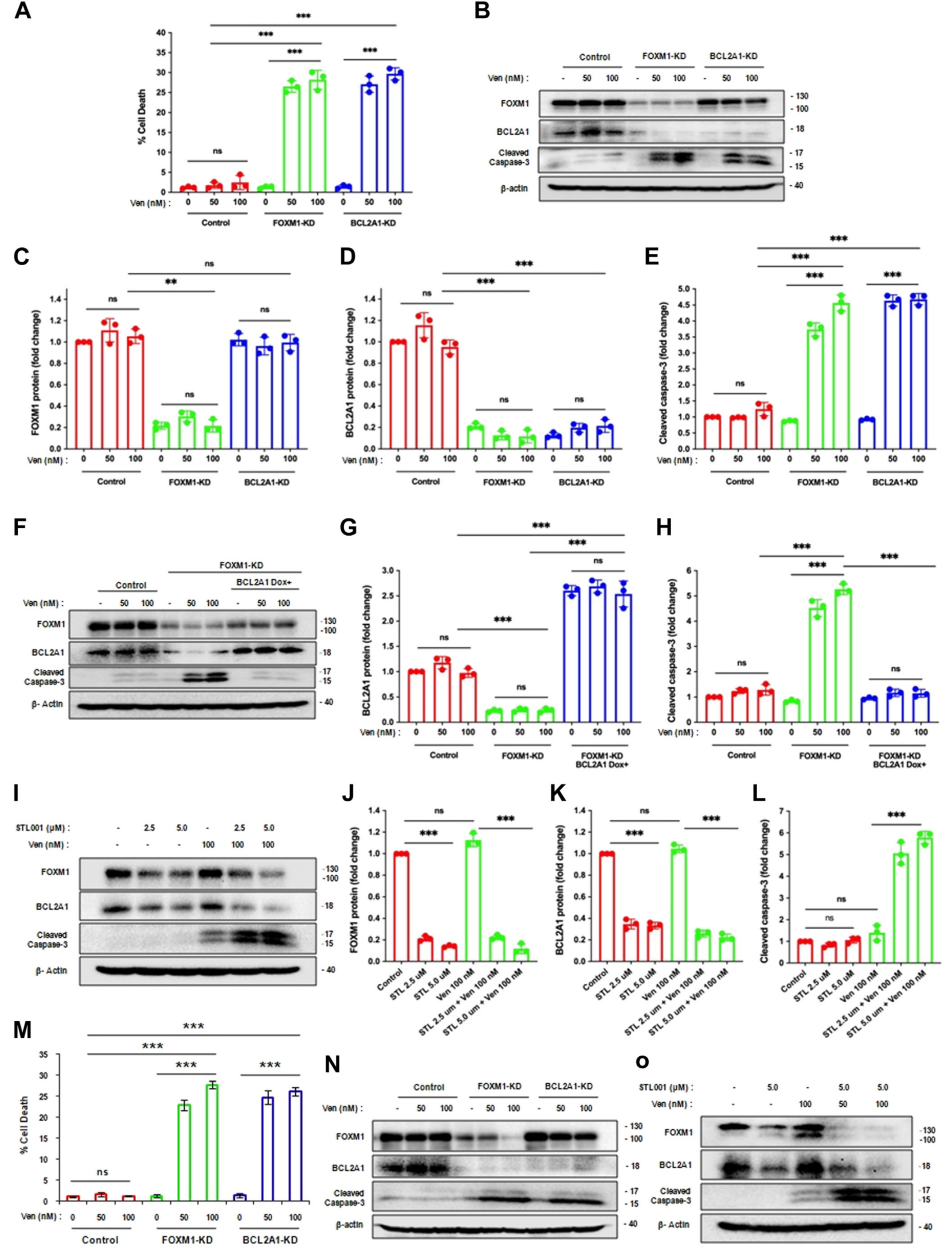
**FOXM1-mediated stabilization of BCL2A1 expression is sufficient to protect cells from BCL2 inhibitor venetoclax (Ven)–induced apoptosis.***A*, KG-1 cells with stable shRNA–mediated FOXM1-KD, BCL2A1-KD, or control vector were treated with increasing concentrations of Ven (0, 50, and 100 nM). The percentage of dead cells was determined *via* a trypan *blue* exclusion test, n = 3 biologically independent replicates. *B*, KG-1 cells with stable shRNA–mediated BCL2A1-KD, FOXM1-KD, or control vector were treated with increasing concentrations of Ven (0, 50, and 100 nM). In all cases, total protein samples were obtained from cells immediately after treatment and analyzed for FOXM1, BCL2A1, and cleaved caspase-3 levels *via* immunoblotting, and β-actin was used as internal loading control. *C*–*E*, densitometry quantitation of bands in three independent experiments related to *B*. *F*, FOXM1-KD-KG-1 cells with doxycycline (Dox)-inducible BCL2A1 expression lentivirus, FOXM1-KD, or control vector were treated with increasing concentrations of Ven (0, 50, and 100 nM). In all cases, total protein samples were obtained from cells immediately after treatment and analyzed for FOXM1, BCL2A1, and cleaved caspase-3 levels *via* immunoblotting, and β-actin was used as internal loading control. *G* and *H*, densitometry quantitation of bands in three independent experiments related to *F*. *I*, KG-1 cells were treated with indicated concentrations of Ven alone or in combination with STL001 for 24 h. In all cases, total protein samples were obtained from cells immediately after treatment and analyzed for FOXM1, BCL2A1, and cleaved caspase-3 levels *via* immunoblotting, and β-actin was used as internal loading control. *J*–*L*, densitometry quantitation of bands in three independent experiments related to *I*. *M*, HL-60 cells with stable shRNA–mediated FOXM1-KD, BCL2A1-KD, or control vector were treated with increasing concentrations of Ven (0, 50, and 100 nM). The percentage of dead cells was determined *via* a trypan *blue* exclusion test, n = 3 biologically independent replicates. *N*, HL-60 cells with stable shRNA–mediated BCL2A1-KD, FOXM1-KD, or control vector were treated with increasing concentrations of Ven (0, 50, and 100 nM). In all cases, total protein samples were obtained from cells immediately after treatment and analyzed for FOXM1, BCL2A1, and cleaved caspase-3 levels *via* immunoblotting, and β-actin was used as an internal loading control. The blot shown is representative of two independent experiments with consistent results. *O*, HL-60 cells were treated with indicated concentrations of Ven alone or in combination with STL001 for 24 h. In all cases, total protein samples were obtained from cells immediately after treatment and analyzed for FOXM1, BCL2A1, and cleaved caspase-3 levels *via* immunoblotting, and β-actin was used as an internal loading control. The blot shown is representative of two independent experiments with consistent results. *B*, *F*, *I*, band intensities were quantified using ImageJ software; data are normalized to “Control/0 nM” sample; all data are presented as mean ± SEM; n = 3 biologically independent replicates; comparison of mean values between multiple groups was evaluated by one-way ANOVA; two-way ANOVA was used to compare groups with two independent variables (∗∗*p* < 0.002, ∗∗∗*p* < 0.001, ns, nonsignificant difference). FOXM1, Forkhead box M1; KD, knockdown.

Further, to investigate if BCL2A1 suppression alone is sufficient to sensitize AML cells to venetoclax, we performed a trypan blue dye exclusion test and immunoblot analysis of KG-1 cells with stable shRNA–mediated FOXM1-KD, BCL2A1-KD, or control vector and dose gradient of venetoclax. BCL2A1-KD completely recapitulated the effect of FOXM-KD and demonstrated a 15- to 20-fold increase in cell mortality compared with control vector cells (Fig. 2*A*). Immunoblot analysis of BCL2A1-KD demonstrated a significant loss of BCL2A1 (Fig. 2, *B* and *D*) with no significant change in FOXM1 levels when compared with control cells (Fig. 2, *B* and *C*). The suppression of either FOXM1 or BCL2A1 resulted in comparable sensitization to venetoclax, as indicated by caspase-3 cleavage (Fig. 2, *B* and *E*), whereas control cells maintained relatively constant FOXM1 and BCL2A1 levels (Fig. 2, *B*–*D*) and full resistance to venetoclax up to 100 nM (Fig. 2, *B* and *E*). Nevertheless, suppression of BCL2A1 in the presence of high levels of FOXM1 was sufficient to sensitize AML cells to venetoclax-induced apoptosis (Fig. 2*B*), suggesting that BCL2A1 is a major target of FOXM1 that inhibits venetoclax-stimulated apoptosis in AML.

To confirm these findings, trypan blue dye exclusion test and immunoblot analysis experiments were repeated using HL-60 cells with stable shRNA–mediated FOXM1-KD, BCL2A1-KD, or control vector and dose gradient of venetoclax (Fig. 2, *M* and *N*). Venetoclax treatment significantly increased mortality rates for FOXM1-KD- or BCL2A1-KD-HL-60 cells (∼20-fold) compared with control vector cells (Fig. 2*M*). Consistent with the data from KG-1 cells (Fig. 2*B*), KD of FOXM1 in HL-60 cells demonstrated a trend of reduced BCL2A1 and increased sensitivity to venetoclax, as indicated by caspase-3 cleavage (Fig. 2*N*). Again, KD of BCL2A1 also sensitized HL-60 cells to venetoclax without affecting FOXM1 protein levels (Fig. 2*N*), indicating that BCL2A1 is likely the primary target associated with FOXM1-mediated venetoclax resistance. Our results suggest that the FOXM1–BCL2A1 axis is responsible for venetoclax resistance in AML.

To investigate if Dox-inducible BCL2A1 overexpression inhibits venetoclax-caused cell death, we overexpressed BCL2A1 in FOXM1-KD cells, which are sensitive to venetoclax (Fig. 2*F*). While venetoclax at 50 and 100 nM concentrations strongly induced apoptosis in FOXM1-KD cells (Fig. 2, *F* and *H*), BCL2A1 overexpression in FOXM1-KD cells (Fig. 2, *F* and *G*) completely restored the resistance of these cells to venetoclax-induced apoptosis (Fig. 2, *F* and *H*). These results further support the notion that BCL2A1 is the major downstream effector of FOXM1 that modulates inhibition of venetoclax-stimulated apoptosis.

We have also explored if the pharmacologic inhibition of FOXM1 recapitulates the genetic suppression of FOXM1. We inhibited FOXM1 with STL001, a selective FOXM1 inhibitor, recently studied in our laboratory (25, 26). Indeed, treatment of KG-1 cells with STL001 led to significant suppression of both FOXM1 and BCL2A1 expression (Fig. 2, *I*–*K*). As expected, STL001 did not exert prominent cytotoxic action on its own, but when used in combination with venetoclax, led to potent induction of apoptosis (Fig. 2, *I* and *L*). We further validated that BCL2A1 suppression exhibited a strong correlation with pharmacological inhibition of FOXM1 and sensitized HL-60 cells to venetoclax-induced apoptosis (Fig. 2*O*). The pharmacological results further confirm that BCL2A1 is the primary target of FOXM1 that reduces the sensitivity of AML cells to venetoclax and suggests that pharmacological inhibition of FOXM1 can induce venetoclax-prompted apoptosis in AML cells.

A recent analysis of patient samples from the Beat AML cohort that had been subjected to venetoclax screening identified BCL2A1 as one of the major drivers that affect venetoclax sensitivity (9). We determined earlier that inhibition of FOXM1 also sensitizes AML cells to venetoclax (25). However, the correlation between FOXM1-induced and BCL2A1-induced inhibition of venetoclax has not been previously explored. Here, we show that inhibition of venetoclax-induced cell death by FOXM1 is linked to the expression of BCL2A1. Because FOXM1 is overexpressed in AML, these results suggest that FOXM1-dependent resistance to venetoclax is determined by BCL2A1. These data will help to utilize novel approaches for the treatment of human cancer, particularly AML.

## Experimental procedures

### Cell cultures

The study was conducted with human AML cell lines KG-1 and HL-60 obtained from the American Type Culture Collection. Cells were maintained in Iscove’s modified Dulbecco’s medium supplemented with l-glutamine (2.0 mM), 10% fetal bovine serum, and 1% penicillin–streptomycin (Thermo Fisher Scientific). Cells were grown at 37 °C in a humidified incubator with 5% CO_2_ and confirmed to be mycoplasma negative by routine testing with an in-house PCR method.

STL001 (Vitas-M Laboratory) and venetoclax (Selleck Chemicals) were dissolved in dimethyl sulfoxide (MilliporeSigma). Dox (LKT Laboratories), puromycin (MilliporeSigma), and geneticin (Gold Biotechnologies) were dissolved in sterile water.

### Stable FOXM1-expression KD

KG-1 and HL-60 cells at 70 to 80% confluency on a 12-well tissue culture plate were transduced with the MISSION lentiviral particle carrying pLKO.1-puro vector encoding shRNA against human-FOXM1 transcripts, pLKO.1-NEO shRNA against human-BCL2A1 transcripts, or pLKO.1-puro Non-Target shRNA Control (MilliporeSigma) at a multiplicity of infection of 10 in the presence of Polybrene (10 μg/ml). Transduction was done overnight at 37 °C in a humidified incubator with 5% CO_2_. Transduced cells were selected by their cultivation with antibiotics for 10 days. Puromycin was used at 1.0 μg/ml for the selection of pLKO.1-puro-transduced cells; geneticin (G418 Sulfate) was used at 500 μg/ml for the selection of pLKO.1-NEO-transduced cells. An untransduced negative control plate was used to determine when the selection was complete. Experiments were performed in cells from early passages after freezing.

### Lentivirus production, transduction, and antibiotic selection

The lentiviral pLV[Exp]-EGFP/Neo-TRE>hBCL2A1[NM_004049.4] vector was purchased from Vector builder (Vector ID: VB240228-1448nxa); the tetracycline-inducible lentiviral pCW57.1-hFOXM1b vector was a gift from Adam Karpf (Addgene plasmid #68811). For lentivirus production, human embryonic kidney 293 FT cells were reverse transfected onto poly-l-lysine (Sigma)–coated 10 cm plates. ViraPower Lentiviral Packaging Mix (Invitrogen; 9 μg), 3 μg of lentiviral plasmid with 36 μl of Lipofectamine 2000 reagent (Life Technologies) was packaged in 3 ml of optiMEM, according to the manufacturer's instructions. Viral supernatant was collected from the culture plate, centrifuged at 500*g* for 5 min, and passed through a 0.45 μm filter (Millipore).

KG-1 cells were seeded on 12-well tissue culture plates to achieve ∼40% confluency. The next day, cells were transduced with lentiviral supernatant (2–4 ml) diluted in 2 to 4 ml of complete media with 10 μg/ml polybrene (Sigma). Transduction was done overnight at 37 °C in a humidified incubator with 5% CO_2_. Transduced cells were selected by their cultivation with antibiotics for 10 days. Puromycin was used at 1.0 μg/ml for the selection of hFOXM1b vector–transduced cells; geneticin (G418 Sulfate) was used at 500 μg/ml for the selection of hBCL2A1 vector–transduced cells. An untransduced negative control plate was used to determine when the selection was complete. Experiments were performed in cells from early passages after freezing.

### Drug treatment of cultured cells

Cells were grown in a humidified CO_2_ incubator and harvested by centrifugation at 200*g* for 5 min, and live and dead cells were counted by hemocytometer using 0.4% Trypan Blue (Thermo Fisher Scientific). In different experimental conditions, cell treatment was performed by resuspending cells (1.5 × 10^6^ viable cells/well of a 6-well tissue culture plate; Thermo Fisher Scientific) in a growth medium containing selected concentrations of drugs. Control samples were treated with vehicle only (vehicle concentration <0.3%). After 24 h of incubation, all samples were harvested by centrifugation at 200*g* for 5 min, washed with cold PBS and processed for qRT–PCR and immunoblotting.

### Quantitative real-time PCR

Total RNA was isolated from cells and purified using TRIzol reagent (Thermo Fisher Scientific) and PureLink RNA Mini Kit (Thermo Fisher Scientific) with additional on-column DNAse treatment according to the manufacturer’s instructions. RNA samples were quantified using NanoDrop, and 1 μg of total RNA was reverse transcribed using RevertAid First Strand cDNA Synthesis Kit (Thermo Scientific). The first-strand complementary DNA was used as a template in real-time PCR, using the PowerUp SYBR Green Master Mix (Thermo Fisher Scientific) and Applied Biosystem detection system (ViiA 7 Real-Time PCR System). The cycling program was set as follows: denaturing at 95 °C for 10 min, followed by 40 cycles of 95 °C for 15 s and 60 °C for 1 min. All samples were run in triplicate, and all gene expression data were normalized to *GAPDH*. The primers were designed using Integrated DNA Technology and used to detect human *BCL2A1* (forward: TAAGGCAAAACGGAGGCTGG, reverse: AGGCCGGTTTCACAATATGGA), human *GAPDH* (forward: CCATCTTCCAGGAGCGAG, reverse: CTTGAGGCTGTTGTCATACTTC).

### Protein immunoblotting

An equal number of cells were pelleted and lysed in ice-cold radioimmunoprecipitation assay buffer (Thermo Fisher Scientific) and protease inhibitor cocktail (Thermo Fisher Scientific) with brief sonication. The protein content in each sample was quantified using the Bio-Rad Protein Assay (Bio-Rad). An equal amount of whole-cell lysate (30–40 μg) was separated on 6 to 12% SDS-PAGE and transferred to Immobilon-PSQ PVDF membrane (MilliporeSigma). Membranes were blocked using 5% bovine serum albumin and incubated overnight with specific primary antibodies (FOXM1, BCL2A1, Cleaved caspase-3, all from Cell Signaling Technology; β-actin, from Invitrogen), followed by 1 h incubation with horseradish peroxidase-conjugated secondary antibodies. SuperSignal West Pico PLUS chemiluminescent substrate (Thermo Fisher Scientific) was used for detection using ChemiDoc MP System (Bio-Rad). Protein band quantitation was performed using ImageJ software (National Institutes of Health).

### Statistical analysis

All data are presented as the mean ± SEM of three independent biological replicates. When comparing two groups, an unpaired two-tailed Student’s *t-*test was performed. When comparing more than two groups, one/two-way ANOVA was used. The PRISM was used to perform all statistical analyses and graphical presentation of the data. The immunoblot images shown in the article represent the results of at least three independent biological replicates. Statistical significance was defined as *p* < 0.05.

## Data availability

The authors confirm that the data supporting the findings of this study are available within the article.

## Conflict of interest

The authors declare that they have no conflicts of interest with the contents of this article.
